# The trauma of the tundra tongue: an experimental and computational study of lingual tissue damage following adhesion to a cold metal lamp post

**DOI:** 10.1186/s13005-025-00581-y

**Published:** 2026-01-04

**Authors:** Anders Hagen Jarmund, Ståle Hagen Jarmund, Sofie Eline Tollefsen, Baard Cristoffer Sakshaug, Sverre Helge Torp, Håkon Jarand Dugstad Johnsen, Rita de Sousa Dias

**Affiliations:** 1 Department of Clinical and Molecular Medicine (IKOM), NTNU – Norwegian University of Science and Technology, Postbox 8905, Trondheim, NO-7491 Norway; 2 Department of Mechanical and Industrial Engineering (MTP), NTNU – Norwegian University of Science and Technology, Trondheim, Norway; 3 Department of Pathology, St. Olavs Hospital, Trondheim University Hospital, Trondheim, Norway; 4 Department of Physics (IFY), NTNU – Norwegian University of Science and Technology, Trondheim, Norway

**Keywords:** Tundra tongue, Tissue injury, Cold injury, Experimental study, Computer simulation

## Abstract

**Background:**

Tissues can adhere to cold metal surfaces, causing tissue damage upon detachment. For children, this often involves the tongue, so-called tundra tongue. This study aimed to assess the risk and extent of tongue tissue damage following adhesion to cold metal lamp posts.

**Methods:**

Eighty-four porcine tongues were acquired shortly after slaughter. The apex and basis of each tongue were separately brought into contact with a section of a cold metal lamp post and detached gradually or rapidly. Detachment force was recorded and tongues were visually inspected for macroscopic injuries. Selected tongues were assessed histologically. Finite element simulations were conducted in COMSOL Multiphysics to model intra-tissue temperature over time. Each tongue was tested twice (apex and basis), resulting in 168 experiments. Four experiments were excluded, leaving 164 for analysis.

**Results:**

Avulsion injury occurred in 89 of 164 experiments (54%) with risk increasing with the detachment force (*p* < 0.001). Peak tongue detachment force was associated with contact time (*p* < 0.001), tongue region (apex versus basis, *p* < 0.01), and release type (rapid versus gradual, *p* < 0.001). A non-linear relationship was found between metal temperature and detachment force (*p* < 0.01), with peak adherence at approximately −7.5 °C. Histological evaluation demonstrated intra-epidermal damage that could be rationalized with computer modelling. Nearly all experiments (92%) caused macroscopic, but not microscopic, cold injury, with risk increasing at lower metal temperatures. Computer simulations suggest that tissue damage is related to superficial tissue freezing that occurs under typical winter conditions and short exposure times.

**Conclusions:**

The tundra tongue is a complex phenomenon. Although the risk of avulsion injury is significant, our findings indicate limited damage potential under normal winter conditions and brief exposure times.

**Supplementary Information:**

The online version contains supplementary material available at 10.1186/s13005-025-00581-y.

## Background

Licking cold metal surfaces in winter and becoming stuck is a surprisingly common yet understudied phenomenon. To our knowledge, only two prior studies have addressed this topic: a case study by Stough, who coined the term *tundra tongue* after his son adhered his tongue to a freezer [1], and a recent scoping review of cases reported in Scandinavian newspapers [2]. Although long-term consequences are seemingly rare, serious complications have been reported, often requiring medical treatment. However, the exact nature of the tissue damage has not yet been systematically studied.

The adhesion of tissue to cold metal is a complex interplay of thermal and mechanical factors. Metals, such as aluminum and steel, have high thermal conductivity, causing rapid heat transfer upon contact. Human saliva, acting as a thin liquid interface, freezes quickly at sub-zero temperatures, forming an ice bridge between the tongue and the metal. The strength of the adhesion depends on multiple factors, such as saliva composition, tongue surface topology, and temperature gradients. The tongue itself is a multilayered organ composed of non- and para-keratinized stratified squamous epithelium (masticatory mucosa) covering connective tissue (lamina propria), which bears structural similarities to the skin’s epidermis and dermis, and intrinsic and extrinsic skeletal muscles [3]. The epithelium is separated from the lamina propria by the basement membrane, with rete pegs anchoring the epithelium to the underlying connective tissue [4]. Histologically, the epidermis can be divided into (from outwards to inwards) strata corneum, granulosum, spinosum, and basale. Friction blisters typically form intra-epidermally in the stratum spinosum with stratum granulosum as roof [5], while suction blisters often occur at the dermal-epidermal junction with basal membrane as roof [6], suggesting that both interfaces are structurally weak, although the blister dynamics likely differ from the tundra tongue scenario. The distribution of fat and muscles differs within the tongue, with the apex being more muscular while the basis contains more fatty tissue, although this is subject to large variation between individuals and age [7]. Epithelial furrows and papillae, particularly on the dorsal surface, may serve as natural anchoring points for ice, enhancing adhesion. Ice formation within the tongue tissue may occur at varying depths, depending on metal temperature and exposure time, potentially leading to cold injury. Forceful detachment of the tongue from the metal surface may result in avulsion injuries, likely influenced by the depth of tissue freezing and the mechanical properties of the ice bridge, but the association between these factors remains unknown.

Computer simulations and ex vivo experiments are useful approaches to studying these damage mechanisms when human studies are ethically challenging. While several commercial options exist for bioheat simulation, COMSOL Multiphysics^®^ is widely utilized for modelling coupled physical systems via the finite element method. A range of studies have employed COMSOL to examine heat and cold in biological tissues [8–14]. However, computational models alone cannot fully capture the complex biological response of tissue to freezing and mechanical detachment. To understand the anatomical and biomechanical effects, biological models are required. The porcine tongue serves as an attractive study model due to its anatomical similarities to the human tongue; it is a suitable substitute for pharmaceutical research [15], and porcine oral soft tissue is biomechanically comparable to humans [16]. Combined, these experimental and computational methods provide a powerful, multi-scale approach to understanding how lingual tissue responds to freezing contact. In this study, we aimed to map the extent of lingual tissue damage following contact with cold metal surfaces under controlled conditions. We hypothesized that tissue damage correlates with detachment force, which in turn can be predicted based on application time, metal surface temperature, tongue region (apex versus basis), and detachment method (gradual versus rapid release). Systematic experiments were conducted with porcine tongues to evaluate both macroscopic and microscopic damage, complemented by computer simulations to model the microscale impact of metal temperature and exposure time.

## Methods

### Materials

Porcine tongues were collected from domestic pigs (species: *Sus scrofa domesticus*, Norsk landsvin) shortly after slaughter at a licensed slaughterhouse and kept cold. Later, on the same day, the tongues were grouped into batches of six and heated to approximately 37 °C in a water bath. Each tongue was assigned to two experimental conditions (apex and basis), with each region randomly assigned to either rapid or gradual release using block randomization (https://www.random.org/lists/). Tongues were tested in fixed order within each batch. Results are not presented by sex as this information was not recorded by the slaughterhouse.

### Experimental procedures

We conducted a laboratory-based intervention study. The adhesion experiments were conducted on three separate days in March 2025. The data were collected as part of a master thesis project [17].

The tongues were mounted at the tongue base to a force sensor (Strain gauge load cell and NAU7802/HX711, Adafruit, New York, USA) for mass measurement. The area of interest (apex or basis) was lubricated with researcher-donated saliva and manually pressed against a cold metal surface. After brief contact, detachment from the metal surface was performed either gradually using a stepper motor (DM320T stepper driver and 17HE19-2004 S stepper motor, StepperOnline, https://www.omc-stepperonline.com/) at 50 mm s^− 1^ or rapidly via manual jerk. Application time and a brief delay preceding release were determined by an operator to ensure sufficient variation in contact time for meaningful statistical analysis. The procedures are demonstrated in Supplemental Video 1.

Maximum head retraction velocity was estimated prior to the experiments to provide a realistic parameter for a human rapidly detaching from a lamp post. Video analysis of manual head retraction indicated a peak velocity of 1.5 m s^− 1^, which could not be replicated by the stepper motor, justifying the use of manual jerk to simulate a rapid release.

**Fig. 1:**
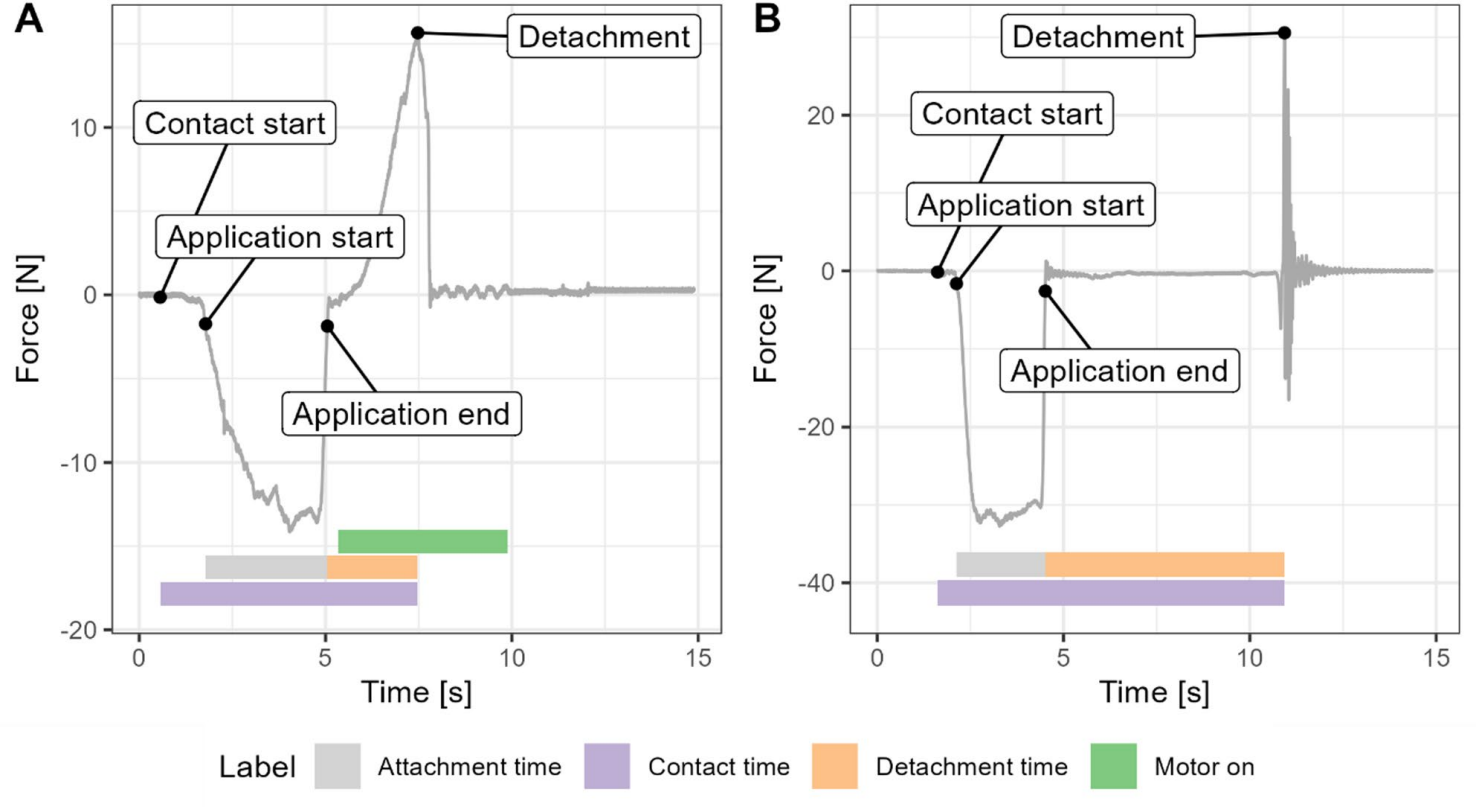
Force-time curves. Representative force-time curves from tongue detachment experiments, using (**A**) gradual release with a motor and (**B**) rapid release by manual jerk. The force exerted on the metal lamp post was measured throughout tongue attachment and detachment. The curves were used to define the duration of distinct experimental stages, as indicated, and to determine the peak detachment force

**Fig. 2:**
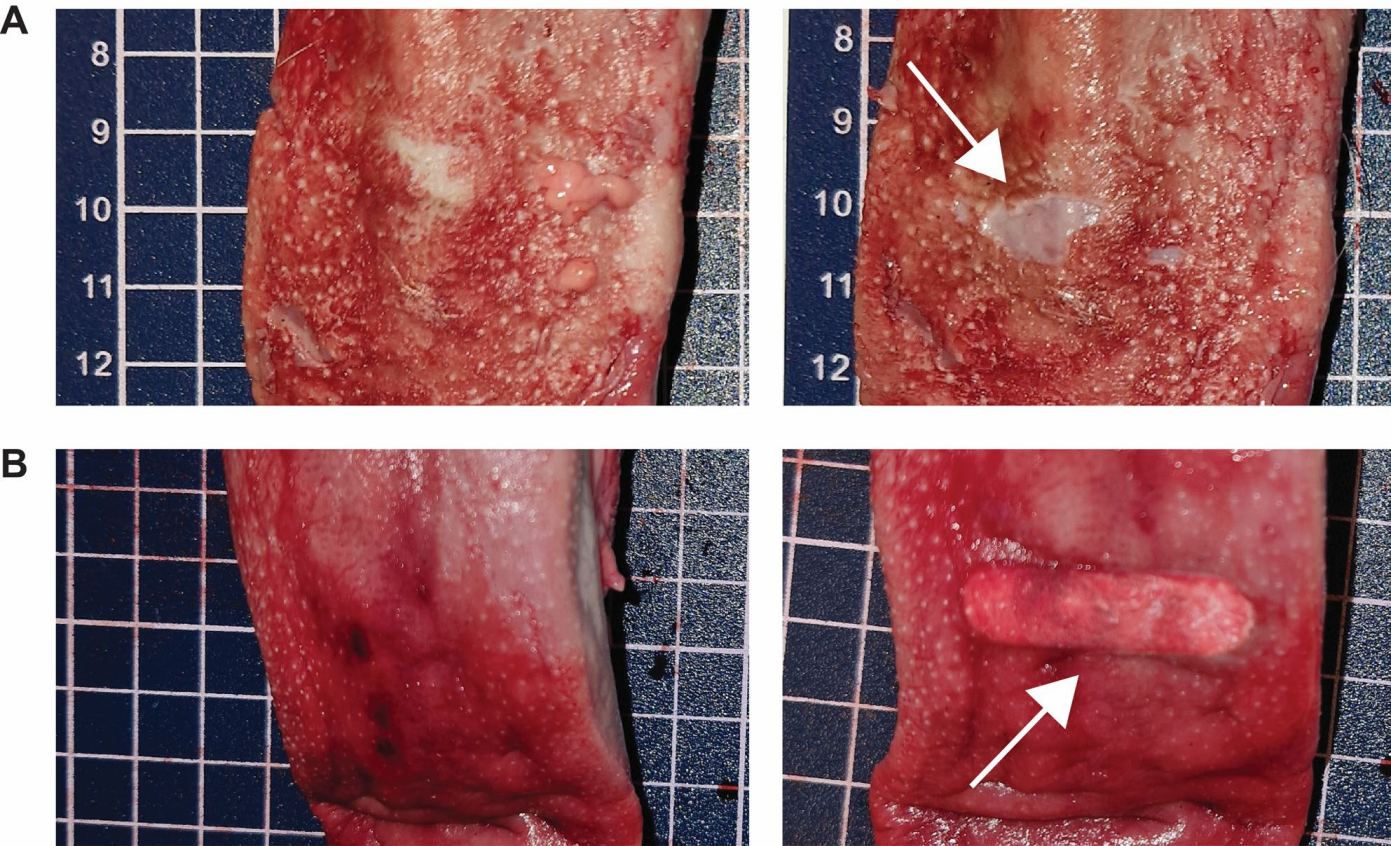
Example of tongue tissue damage following detachment, showing (**A**) avulsion and (**B**) cold injury, indicated by white arrows. Left-hand panels show the tongue surface before the experiment, and right-hand panels show the same regions after detachment. In B, the cold injury has a well-defined boundary corresponding to the oval opening of the polylactic acid (PLA) template shown in Figure S1. Squares in the background are 1 × 1 cm

During attachment and detachment, the force exerted on the metal surface was recorded at 320 Hz using a force sensor (Strain gauge load cell and NAU7802/HX711, Adafruit, New York, USA). Figure 1 shows representative force profiles during gradual and rapid release experiments, highlighting how attachment, detachment, and contact times are defined in this work. Key components of the experimental setup are shown in Figure S1. Each tongue was visually inspected and photographed before and after each experiment. Aversion and cold injury were scored as either present or absent by visual inspection of the tongues (Fig. 2).

The tongues were adhered to a cooled 250 mm long section of a representative Norwegian streetlight (Vik Ørsta AS, Norway), composed of hot-dip galvanized S355 structural steel. The streetlight section was cooled to approximately −40 °C using dry ice. The section had an inner diameter of 107.1 mm and a wall thickness of 3.6 mm. To standardize the contact area, the metal surface was covered with a 0.4 mm-thick polylactic acid (PLA) template featuring elongated oval openings measuring 30 × 10 mm.

Metal surface and tongue temperatures were measured using an infrared (IR) camera (MLX90640, Adafruit, New York, USA), capturing 24 × 32 pixel images at 1 Hz. The emissivity was adjusted from 0.95 to 0.92 to account for the PLA coating [18]. Metal temperature was estimated using the mean of the 20 lowest pixel values. A thermocouple (DS18B20, Adafruit, New York, USA) validated the metal surface temperature. Ambient conditions (temperature, pressure and humidity) were recorded continuously using a BME688 sensor (Adafruit, New York, USA).

### Histology

Selected tongues were fixed in formalin for two weeks, embedded in paraffin, sectioned, and stained with hematoxylin, eosin, and saffron (HES) following standard protocols. Whole-slide brightfield images were scanned using an Olympus Slideview VS200 system with a 20× objective. The procedures were conducted at the Cellular & Molecular Imaging Core Facility (CMIC), NTNU, by an experienced senior bioengineer.

### Computer modelling

Finite element simulations using COMSOL Multiphysics have been widely applied to study temperature distribution and evolution in biological tissues, including cancer tumors [12], temperature rise from humeral bone nails during magnetic resonance imaging [11], gold nanoparticle enhanced photothermal therapy [13], tissue cauterization in endovenous laser coagulation [14], and cryosurgery [8–10]. In the latter, simulation results have been experimentally validated. Most of these studies employ Pennes’ bioheat equation to describe heat transfer within tissue, accounting for conduction, convection, and blood perfusion. Since the present experiments were conducted using ex vivo tissue and included a non-tissue element (metal), the Heat Transfer in Solids Interface in COMSOL Multiphysics (version 6.0) was used [19].

High-resolution images of porcine and human histological sections were used to develop tongue models. Images were downscaled and segmented manually or using the Segment Anything Model (SAM) [20], followed by manual refining. Segments were post-processed in Adobe Illustrator and exported as DXF files. Segmented images were imported into COMSOL to create a 2D heat transfer model of tongue-metal contact. Thermophysical properties of all components are listed in Table S1.

The initial temperatures were set to 37.0 °C for tongue domains (custom material), 20.0 °C for the saliva layer (water, thickness 0.04–0.1 mm), and measured pipe temperature for the metal (steel AISI 4340, thickness 4.6 mm). Blood vessel regions were assigned a Dirichlet boundary condition of 37.0 °C to simulate perfusion. The system was closed with no heat flux (d*Q* = 0) across outer surfaces. A physics-controlled mesh with “Coarser” element size was generated to balance resolution and computational cost (Fig. S3). Because histological sections were used to construct the tongue models, the geometry implies that the metal pipe conforms to the tongue shape rather than vice-versa. This effect was minimized when a simplified tongue model was considered:

To generalize findings to a pediatric anatomy, a parametric tongue model was developed using the Build123 python library. In this model, tissue layers and interfaces were based on defined parameters rather than directly from the histology. Variation in vessel size and interface roughness was introduced using sinusoidal perturbations.

Simulation times were *t* = 15 s with a fixed time step of 0.1 s. This took approximately 10 min on a MacBook Pro M2 Pro.

### Statistical analysis

Desired sample size (85 tongues, 170 experiments) was calculated before study start using G*Power (version 3.1.9.7; fixed model, single regression coefficient), assuming a linear regression model with four predictor variables (Bonferroni corrected $\:\alpha\:=0.05/4=0.0125$), two-tailed test, small-to-moderate effect size ($\:{\mathrm{f}}^{2}=0.1$), power 0.90, and allowing for up to 15% failed experiments.

A linear mixed model was used to assess the effects of contact time, tongue region (apex versus basis), detachment method (gradual versus rapid), and metal temperature on the detachment force. Tongue (nested within batch) was used as a random intercept. Residuals were visually inspected for normality, and p-values were calculated using Satterthwaite’s method [21].

A logistic binomial regression model applying a logit linking function with random intercept was used to assess the risk of avulsion and cold injury. For avulsion injury, two models were compared: one including the four experimental variables (contact time, tongue region, detachment method, and metal temperature), and another using detachment force as the sole predictor. Cold injury was modeled only in relation to the four experimental variables. In both models, tongue (nested within batch) was included as a random intercept to account for repeated measures and potential batch effects.

The linear mixed model described above was used to estimate detachment force across a defined grid of experimental conditions (varying temperatures, contact time, tongue region, and detachment method), while ignoring random effects. The logistic model for avulsion injury was then used to find the associated risk for each estimated detachment force. For cold injury, a similar approach was applied, but the detachment method was excluded from the model. Risk estimates were derived directly from metal temperature, contact time, and tongue region. Missing data was not imputed.

### Patient and public involvement

No patients or members of the public were involved in conception, design, or execution of this laboratory-based study.

### Ethics

This study was conducted according to the principles of the Declaration of Helsinki. The human tongue histology was obtained from a deceased individual that had donated his/her body to the Department of Clinical and Molecular Medicine, Norwegian University of Science and Technology (NTNU), Norway for research and educational purposes. The individual provided informed written consent. No human tissue was used in the detachment experiments. Porcine tongues were purchased from a licensed slaughterhouse (Nortura slakteri, Steinkjer, Norway) via the Farmer’s market (Bondens utsalg) and handled according to university guidelines and animal by-products regulations.

## Results

### Baseline characteristics

A total of 168 experiments were conducted on 84 unique porcine tongues, divided into four experimental conditions (Table 1). Of these, four observations were excluded due to experimental errors such as the peak force occurring outside the sampling window, leaving 164 experiments for analysis. The experimental conditions for each tongue region were comparable in terms of tongue weight and temperature, application time, and apical quality. Ambient variables varied between days (Fig. S2), but not across the experimental conditions (Table 1). The tongue temperature was slightly below the target of 37.0 °C.

**Table 1:** Experimental conditions and outcomes. Ambient conditions and experimental outcomes, divided by tongue region (apex versus basis) and detachment method (gradual versus rapid release)

	Apex			Basis		
Characteristic	Gradual^*a*^	Rapid^*a*^	p^*b*^	Gradual^*a*^	Rapid^*a*^	p^*b*^
Number of experiments, n	40	42		41	41	
Experimental conditions						
Ambient pressure, hPa	997.3 (2.8)	997.4 (2.8)	0.8	997.5 (2.8)	997.2 (2.8)	0.7
Ambient temperature, °C	24.0 (0.2)	24.0 (0.1)	0.8	24.0 (0.2)	24.0 (0.2)	0.8
Application time, s	3.8 (1.8)	3.7 (1.9)	0.7	4.2 (1.8)	4.1 (1.9)	0.8
Metal temperature, °C	-13.8 (8.1)	-13.5 (7.9)	0.9	-15.0 (8.6)	-12.1 (7.9)	0.11
Ambient humidity, %	17.5 (4.4)	17.8 (4.4)	0.7	17.9 (4.5)	17.4 (4.3)	0.6
Poor quality,^*c*^ n	25 (63)	24 (57)	0.7	0 (0)	0 (0)	> 0.9
Tongue weight, g	193.2 (24.9)	199.5 (27.5)	0.3	194.6 (25.6)	197.6 (25.6)	0.6
Tongue temperature, °C	34.7 (2.2)	34.7 (1.7)	0.9	34.5 (2.1)	34.7 (1.6)	0.7
Unknown temperature, n	1	1		0	1	
Experimental outcomes						
Detachment force, N	10.9 (8.3)	16.4 (10.7)	0.011	13.0 (11.3)	22.6 (13.2)	< 0.001
Contact time, s	7.7 (2.2)	6.0 (2.5)	0.001	7.8 (2.1)	5.7 (2.8)	< 0.001
Detachment time, s	3.9 (1.5)	2.3 (1.9)	< 0.001	3.6 (1.8)	1.6 (1.4)	< 0.001
Avulsion injury, n	21 (53)	20 (48)	0.8	21 (51)	27 (66)	0.3
Cold injury, n	36 (90)	39 (93)	0.7	38 (93)	38 (93)	> 0.9

^*a*^Mean (SD); n (%)

^*b*^Welch Two Sample t-test; Fisher’s exact test

^*c*^Poor quality refers to damaged tongue surface from the slaughter process, occurring prior to experiments

### Experimental outcomes

#### Detachment force evaluation

**Fig. 3:**
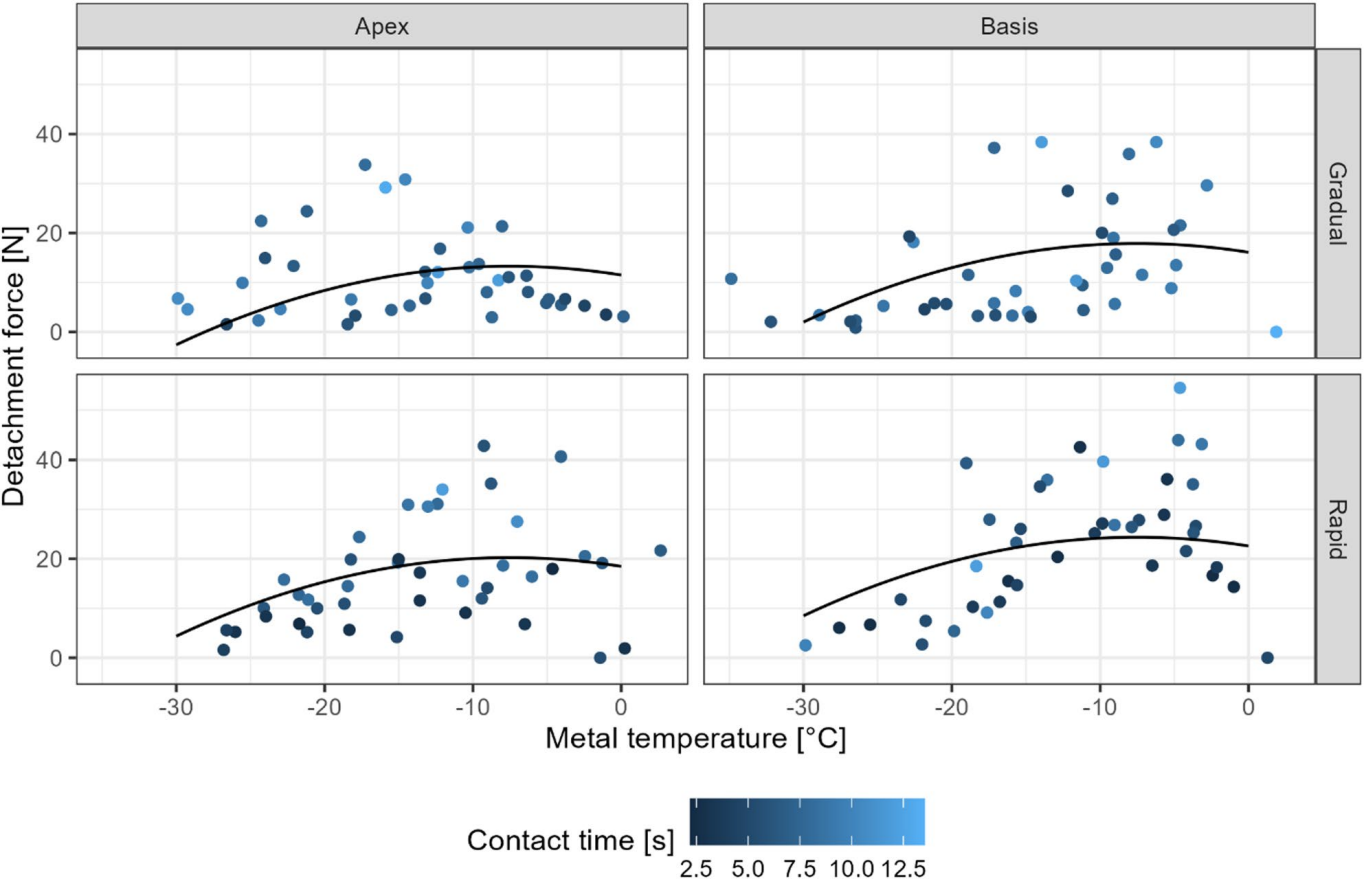
Detachment force of the tongue at varying metal surface temperatures, divided by tongue region (apex versus basis) and detachment method (gradual versus rapid release). Individual data points are shown as blue dots, with darker points indicating longer contact times. Solid lines represent second-order polynomial fit derived from a linear mixed model, adjusted for contact time

The force required to detach the tongue from the cold metal surface is shown in Figure 3 and differed between the various experimental conditions (Table 1). Rapid release required a higher detachment force than gradual release, both at the apex and the basis of the tongue. As anticipated, contact time was strongly correlated to application time ($\:{r}_{\mathrm{s}}=.71$, $\:S=216,016.29$, $\:p<.001$), and their difference was larger for gradual compared to rapid release ($\:\varDelta\:M=1.77$, 95% CI $\:\left[1.26,2.29\right]$, $\:t\left(161.99\right)=6.79$, $\:p<.001$).

Detachment force increased with longer contact time and higher metal temperatures (Model 1 in Table S2). It was greater at the tongue basis than at the apex, and higher for rapid than for gradual release. The unexpected non-monotonic relationship between detachment force and metal temperature warranted further inquiry and a quadratic term was added post-hoc. The quadratic term was highly significant, suggesting a non-linear relationship and indicating a peak detachment force at approximately −7.5 °C (Model 2 in Table S2; Fig. 3). Model 2 was preferable to model 1 according to both likelihood ratio testing (χ²(1) = 9.99, *p* = 0.002) and information criteria (ΔAIC = 8.0, Akaike weight = 0.98).

#### Risk of avulsion injury

**Fig. 4:**
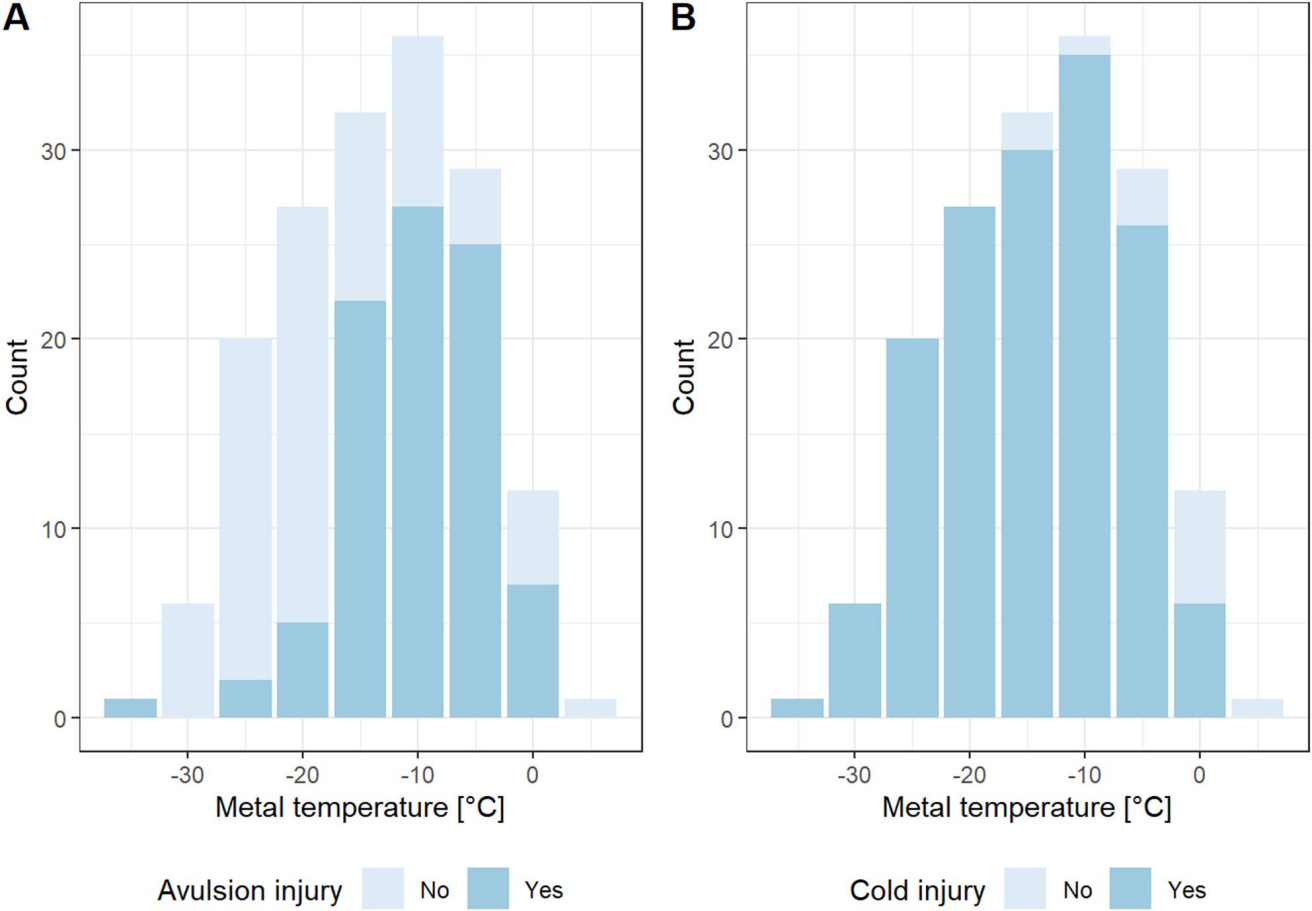
Occurrence of avulsion and cold injuries. Histograms showing the occurrence of (**A**) avulsion and (**B**) cold injuries (*n* = 164)

**Fig. 5:**
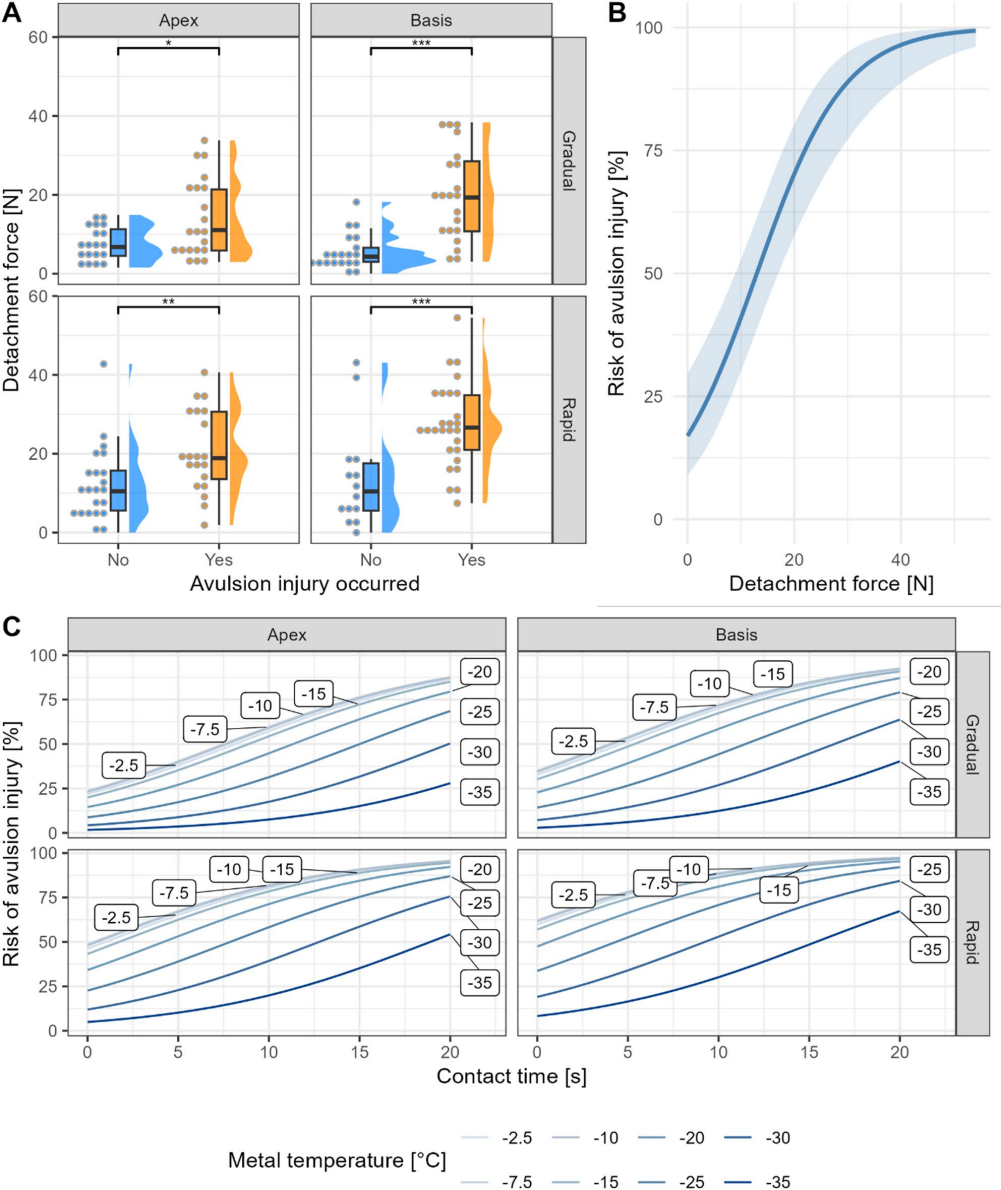
Risk of avulsion injury. **A** Detachment force was significantly higher in cases of avulsion injury across tongue region (apex versus basis) and detachment method (gradual versus rapid release). Binned raw data shown as dots. **B** Overall risk of avulsion as function of detachment force. Ribbons indicate 95% confidence interval. **C** Estimated risk for avulsion injury as calculated from tongue region, detachment method, contact times and metal surface temperatures. P values from Wilcoxon rank-sum test. * *p* < 0.05, ** *p* < 0.01, *** *p* < 0.001

A total of 89 (54%) avulsion injuries were recorded during the experiments (Table 1), predominantly occurring at metal temperatures between −5 °C and −15 °C (Fig. 4A). The risk of avulsion injury was strongly associated with detachment force: for each standard deviation increase in detachment force, the risk of injury increased 323% ($\:\widehat{\beta\:}=1.44$, 95% CI $\:\left[0.90,1.98\right]$, $\:z=5.21$, $\:p<.001$). Figure 5 shows raw data as well as estimated risk based on detachment force, modelled from metal temperature, contact time, detachment method, and tongue region.

Interestingly, the risk for avulsion injuries increased with *increasing* metal temperature (Table S3). However, no significant associations were found with contact time, detachment method (rapid versus gradual release), or tongue region (basis versus apex).

#### Risk of cold injury

Cold injury occurred in most experiments ($\:n=151$, 92%; Table 1; Fig. 4B). The risk increased significantly with decreasing metal temperatures (Table S3), and no injuries were observed at temperatures above 0.2 °C. Due to the low number of non-injured cases, a formal risk model for cold injury was not developed.

### Histological findings

Histological sections were prepared from porcine tongues (14 sections from 7 individuals), including controls (two sections from one individual) and post-exposure samples (12 sections from 6 individuals), as well as from a human tongue (three sections from one individual).

#### Avulsion and cold injuries

Sections from porcine tongues with avulsion injury (*n* = 8 sections) were of good staining quality and suitable for histological evaluation. The depth of the injury was typically located superficially to the basal membrane, in the epidermis.

Although the macroscopic evaluation suggested cold injuries in 92% of porcine tongues, no clear histological signs of cold injury were observed in the corresponding histological sections (*n* = 10 sections).

#### Inter-species comparison

Structural differences in anatomical microscopic structure were noted between the porcine and human tongue tissues. Human tongues exhibited considerably more interstitial fat, less muscle tissue, and a more complex vascular structure compared to porcine tongues.

### Computer simulations

**Fig. 6:**
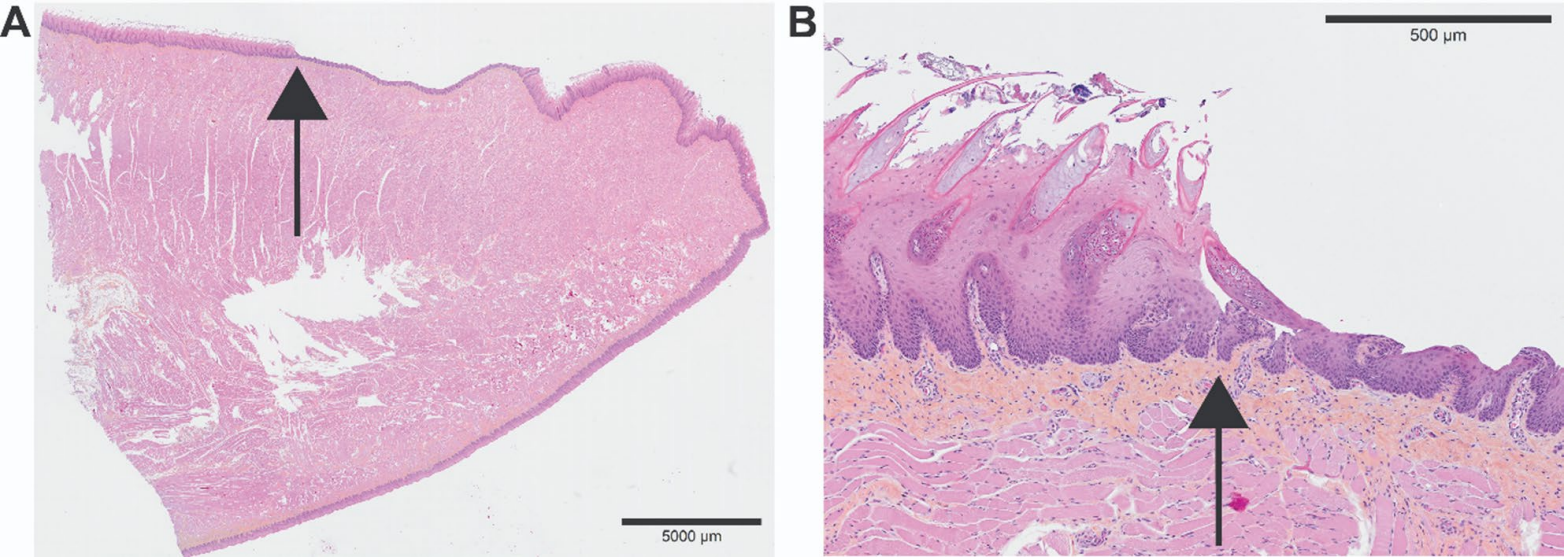
Intra-epidermal avulsion damage. Histological section of a porcine tongue with an avulsion injury following adhesion to a cold metal lamp post at −4 °C. **A** Macroscopic view of the injury site. **B** Microscopic view showing the injury localized intra-epidermally. The black arrow indicates the same anatomical location in both panels. The full-resolution histology image is available at Zenodo: 10.5281/zenodo.17849715

To mechanistically link the experimental data to histological findings, computer simulations were conducted to model tissue temperature dynamics. One high-quality histological image from each species was selected for in-depth analysis; a dorsal apical section from a porcine tongue with avulsion injury (Fig. 6), and a ventral apical section from a human tongue.

#### Avulsion injury in porcine tongues

**Fig. 7:**
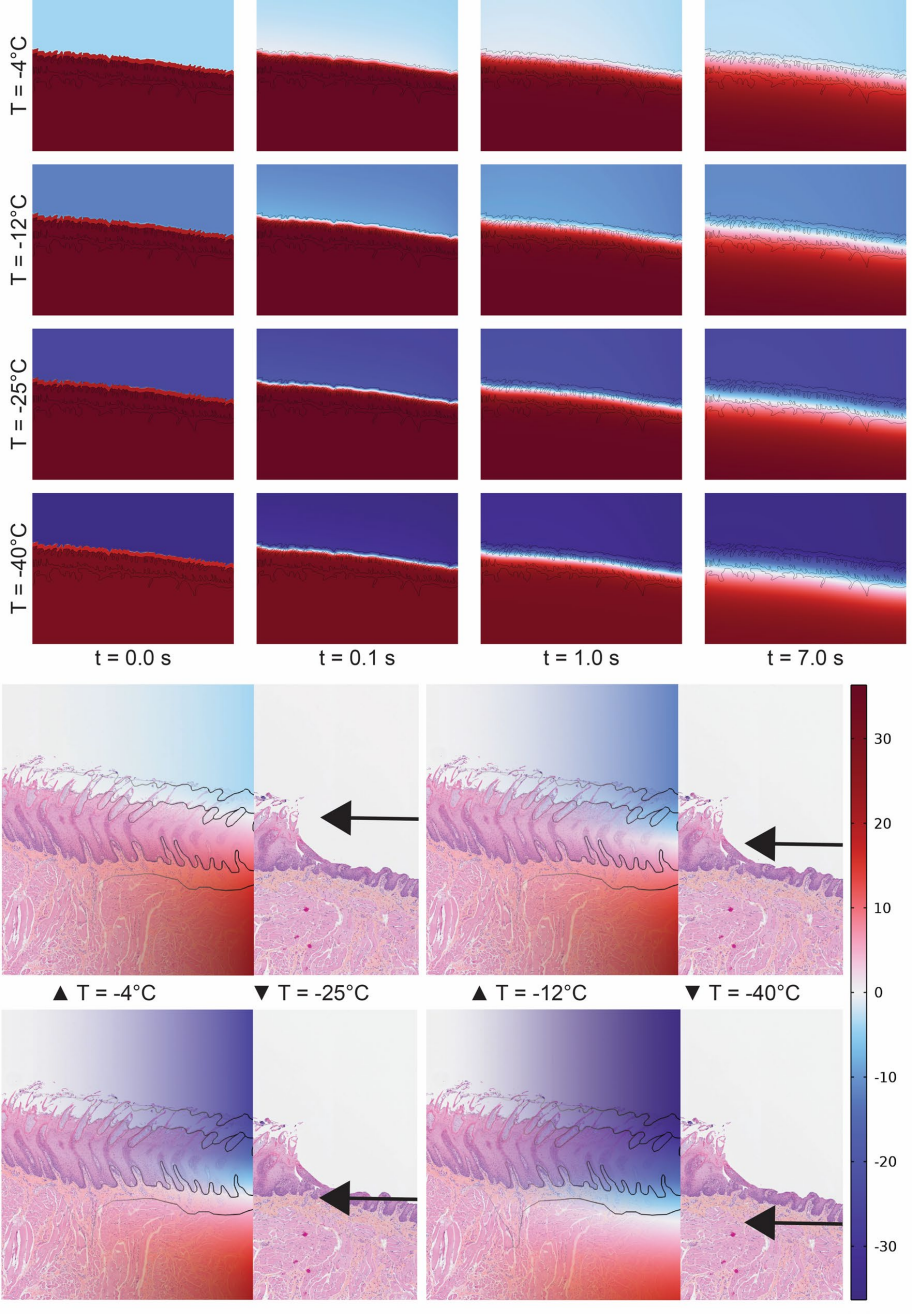
Simulation of tongue freezing dynamics at four metal surface temperatures: −4, −12, −25 and −40 °C. Upper panels show the progression of temperature distribution within the simulated tongue model over time (0–7 s) with decreasing metal surface temperatures (−4, −12, −25 and −40 °C) at baseline. Lower panels show an overlay of the simulated tongue surface at 7 s with the corresponding histological section below (Fig. 6), including the laceration caused by the adhesion experiment (metal lamp post at −4 °C) visualized as a temperature gradient. Black arrows indicate the tissue depth reaching 0 °C, which increases with decreasing metal temperature

**Fig. 8:**
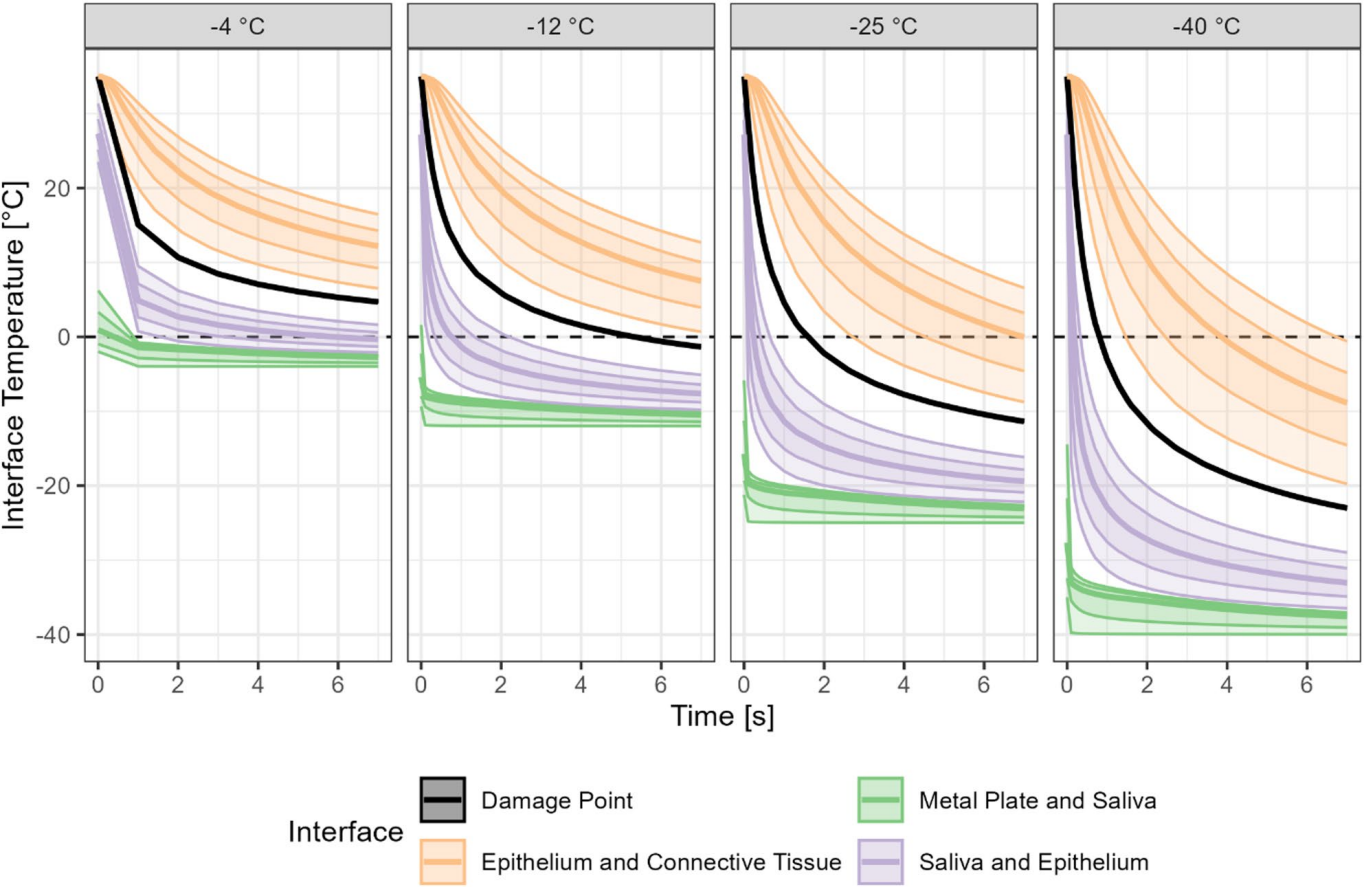
Temperature distribution across various tissue interfaces at different metal temperatures ( −4, −12, − 25, and −40 °C). The damage point, given as a black line, refers to the avulsion depth assessed by histology (Fig. 6). Temperatures were acquired at multiple points along the (rough) interfaces. Bold lines represent median temperature, and shaded ribbons indicate the full range and the 10^th^-90^th^ percentile intervals

Computer simulations show clear differences in the depth of sub-freezing temperatures under various experimental conditions (Fig. 7). They revealed rapid initial cooling of the tissue upon contact with the cold metal, followed by a near-linear decline in tissue temperature after the first few seconds (Fig. 8). As expected, longer contact times and lower metal temperatures led to deeper and colder tissue layers. Interestingly, the depth corresponding to the observed avulsion injury (denoted “Damage point” in Fig. 8) did not reach 0 °C when the initial metal temperature was −4 °C, and only barely did so at metal temperature −12 °C, after 7 s. At −25 °C, parts of the connective tissue reached freezing temperatures within 7 s, unlike at initial metal temperature −12 °C.

#### Predicted avulsion injury in human adult tongues

A histological section of a human adult tongue was used to develop a model (Fig. S4) to study temperature dynamics in adult human tongue tissue during contact with a cold metal surface, as described above. Two models were tested, with and without blood vessels as continuous heat sources (Figs. S5 A and B, respectively). At metal temperature −4 °C, only the superficial epithelium reached sub-zero temperatures within 7 s. At −40 °C, deeper epithelial layer and the connective tissue reached considerably lower temperatures, but only in the model excluding blood flow.

#### Predicted avulsion injury in pediatric tongues

To predict pediatric responses to cold metal surfaces, the tongue model was simplified and parameterized (Fig. S6). This simpler model allowed us to easily customize the thickness and composition of the different tissue layers. Model validation against the segmented porcine model showed comparable results (data not shown).

The pediatric tongue model was developed by reducing adult tongue dimensions by 30%, reflecting the approximate size of a pediatric tongue [22, 23]. The number and dimension of blood vessels were assumed to be similar, and simulations were run with and without vascular heat sources (Figs. S5 C and D, respectively). The relative thickness of the tissue layers was maintained. Parameter values are listed in Table S4.

The predictive pediatric model (Figs. S5 C, D) produced similar results as the segmented adult model (Fig. S5 A, B) but showed lower temperatures in deeper tissue layers. At −4 °C, the superficial epithelium reached sub-zero temperatures, while the connective tissue did not. At extremely cold metal temperatures, the connective tissue reached sub-zero temperatures within 2 s, regardless of blood flow.

## Discussion

**Fig. 9:**
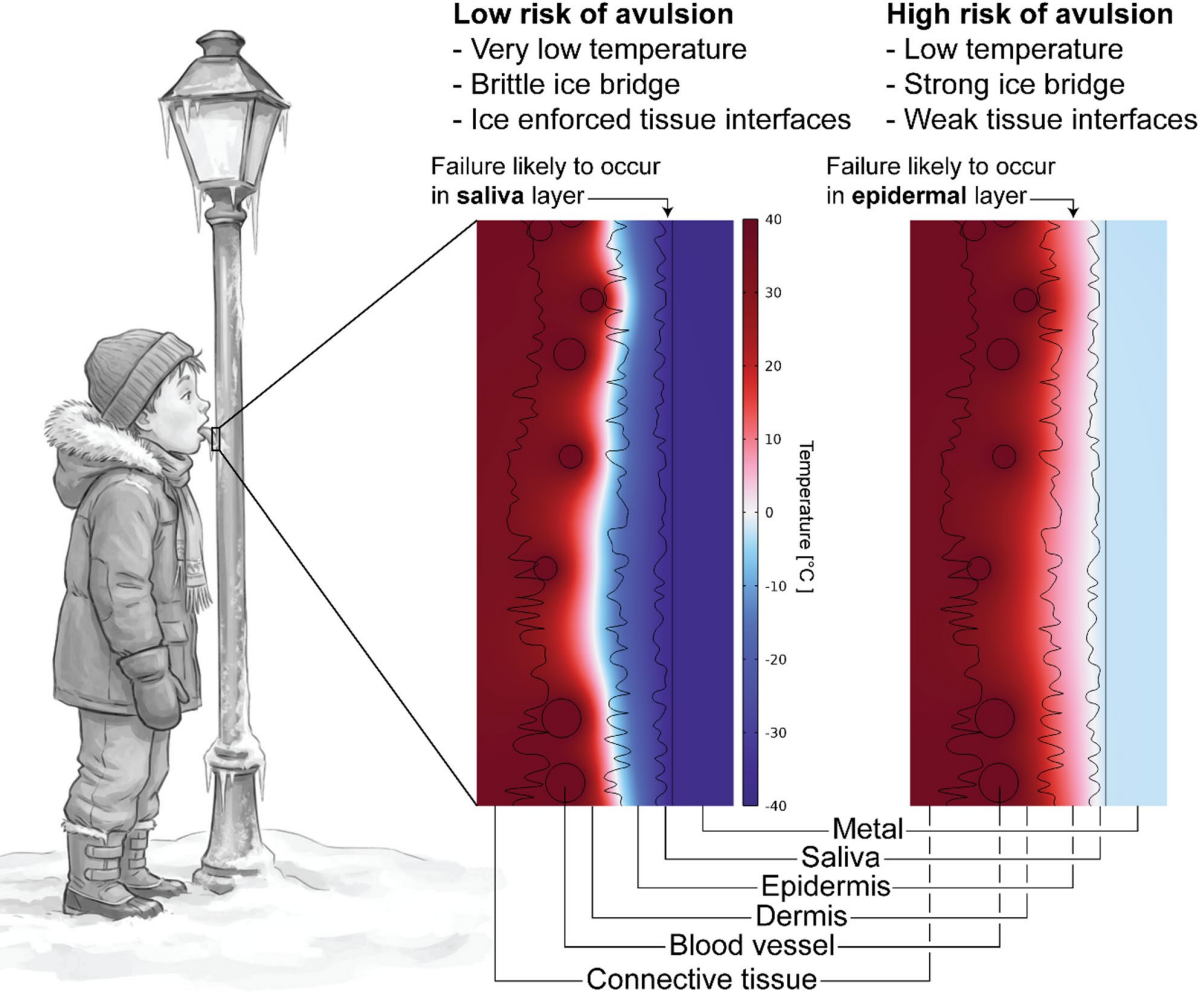
Schematic summary of the main study results. The risk for avulsion injury seems highest at moderately low temperatures (−5 to −15 °C), when the superficial epidermis reaches sub-zero temperatures and leaves the intra-epidermal layers vulnerable for avulsion. At lower temperatures, the risk for avulsion seems lower, possibly due to a combination of changed ice properties and enforced tissue interfaces from freezing, making the saliva layer more likely to break. The left-hand drawing was sketched with generative artificial intelligence (Grok 3, https://grok.com/) and manually refined

In cold climates, children occasionally adhere their tongues to frozen metal surfaces, a phenomenon colloquially known as the “tundra tongue”. This study provides new insights into the mechanisms and risks associated with such incidents (Fig. 9). We have demonstrated that: (i) the risk of avulsion injury peaks at metal temperatures around −7.5 °C, (ii) these injuries are typically intra-epidermal, and (iii) tissue freezing beyond the superficial epithelium is unlikely under normal winter conditions. Additionally, we provide a risk model for avulsion injury incorporating metal temperature, contact time, tongue region and detachment mode. The cross-disciplinary approach used in this study – combining experimental data, histological sections, and computer simulations – offers a robust framework that can be adapted to related biomedical and material science problems.

The adhesion of the tongue to a cold metal surface is commonly attributed to the freezing of saliva and moisture on the tongue, which forms an ice bridge between the two rough surfaces [24]. This phenomenon is driven by the rapid heat transfer from the tongue to the metal, causing localized freezing at the contact interface and forming an ice bridge. The observed non-linear relationship between metal temperature and detachment force was unexpected and highlights the complexity of this process. Several mechanisms may contribute to this non-linear behavior, such as: (i) the properties of the ice bridge, (ii) the ice-metal adhesion, and (iii) the freezing of tongue tissue. Ice exhibits temperature-dependent physical properties, affecting both the strength and brittleness of the ice-tongue interface. The presence of salt ions and proteins in the saliva, and in the tongue tissue, also impacts phase transition dynamics. Formation of ice on metallic surfaces is a well-known challenge in various industries and ice adhesion is a topic of active research in material science [25]. Ice adhesion strength depends not only on the substrate material but also on ice formation pathway, ice properties, and environmental conditions (temperature and humidity). For example, ice adhesion to metals generally increases with decreasing temperature until reaching a plateau [26]. Therefore, the initial increase in detachment force with decreasing temperature can be attributed to stronger ice and enhanced ice-metal adhesion. However, at lower temperatures, the onset of tissue freezing may reduce the effective detachment force due to changes in tissue compliance and tissue interface failure. These two processes may explain the observed experimental results and are consistent with the computer simulation findings (Fig. 9).

Although this is the first experimental study of the tundra tongue specifically, medical adhesive-related skin injuries (MARSIs) offer a useful analogy [27]. These injuries, which occur without tissue freezing, include epidermal stripping (damage limited to the stratum corneum), tension blistering (separation of dermis from epidermis), and skin tears (avulsion of either the epidermis alone, or both the dermis and epidermis). Severity depends on both adhesive and skin properties. While most MARSIs are superficial, limited to the stratum corneum, deeper avulsions have been reported, especially in elderly patients [28–30]. Thinning of the dermal-epidermal junction from aging, and thus increased risk of dissociation at this interface, has been suggested to explain the increased risk of skin avulsion injuries in this age group [29, 30]. Notably, peel force does not always correlate with injury severity [31], highlighting the complexity of avulsion mechanisms. Freezing likely changes tissue mechanical properties, and the structural integrity of the skin, making thawed skin more fragile [32]. Nevertheless, these findings support our observation that tundra tongue injuries are typically intra-epidermal. Cold injuries to other parts of the body, especially the fingers, have also been studied by others. A comprehensive experimental study by Geng et al. [33] found that, for contact with aluminum at −15 °C, the finger skin surface reached 0 °C within 2–6 s, and significantly faster at lower metal temperatures. This is somewhat slower than our computational models predicted, but at comparable order of magnitude.

### Strengths and limitations

This study has several strengths. Experiments were performed under controlled laboratory conditions with standardized protocols and monitoring of ambient variables. Porcine tongues, commonly used in pharmacological testing of drugs intended for human use [15], provided a relevant model due to their anatomical similarity to human tongues.

However, limitations exist. Testing was performed post-mortem, which precludes assessment of bleeding and introduces non-physiological conditions such as absent blood flow – shown in simulations to significantly affect tissue freezing. Tongues were tested shortly after slaughter, minimizing histological degradation [34]. Apical epidermal damage from the slaughter process was common but the consistency of results across tongue regions suggests that the results were still valid.

The use of human saliva adds some validity to the results, though the impact of saliva composition warrants further study. Histological sampling was limited by funding, and the finding of intra-epidermal avulsion injury should be interpreted cautiously. Computer simulations, while informative, are sensitive to model assumptions and parameter values. In this study, simulations were used to explore plausible mechanisms rather than to provide definitive predictions. The exact freezing temperature of tongue tissue is uncertain, but comparable tissues freeze at −0.8 to −1.0 °C [35]. Ethical constraints preclude replication in a human cohort.

### Future research and implications for healthcare

Our findings suggest that the tundra tongue usually is of limited clinical significance but do not rule out the possibility of severe tissue damage. The results suggest that tissue avulsion injuries from the tongue detaching cold metal surfaces, at normal winter temperatures and within a reasonable time, are likely to be limited to the epidermis. While the oral epidermis is well-known to heal rapidly and without scarring [36], the tundra tongue can be painful and distressing. If larger areas are affected, clinical attention may be warranted due to risk of secondary infections, difficulties eating, or need for treatment [2]. Epidermal damage primarily warrants supportive treatment, analogously to superficial intraoral burns [36]. We could not properly assess tissue injury from the cold itself histologically due to post-mortem tissue, and this remains an important area for future research. Computer simulations showed a strong impact from blood flow, demonstrating the importance of including such structures in future ex vivo models; the absence of blood flow in our experimental model may have increased the severity of tissue damage. The impact of the structural design of the lamp post metal surface, including coating and roughness, at very low temperatures deserves further study and may impact policy.

## Conclusions

This study provides new insights into the mechanisms and risks associated with tongue adhesion to cold metal surfaces. Through a combination of controlled experiments, histological analysis, and computer simulations, we demonstrate that avulsion injuries are most likely to occur at moderately low temperatures (−5 to −15 °C) and are typically confined to the mid-epithelium. At extremely low temperatures, the risk of avulsion appears to decrease, possibly due to changes in ice adhesion dynamics and tissue freezing depth. Our findings suggest that while most injuries are superficial and self-limiting, the potential for deeper tissue damage exists under extreme conditions. The interdisciplinary methodology employed in this study offers a valuable framework for investigating other cold-induced tissue injuries and highlights the importance of integrating biological, physical, and computational perspectives in injury research.

## Supplementary Information

Supplementary Material 1.

Supplementary Material 2.

Supplementary Material 3.

## Data Availability

Data are provided within the manuscript or supplementary information files.
