# Supplementary material for: The trauma of the tundra tongue: an experimental and computational study of lingual tissue damage following adhesion to a cold metal lamp post
Source: Head Face Med. 2026 Jan 4;22:12. doi: 10.1186/s13005-025-00581-y (PMC12870143; doi:10.1186/s13005-025-00581-y)
Supplement: Supplementary file 3 — Supplementary Material 3. [file 13005_2025_581_MOESM3_ESM.pdf]

Table S1. Thermophysical properties of modelled components. Tissue properties are based on the IT'IS Tissue Database (1), and metal properties are based on COMSOL defaults for steel AISI 4340.

**Component**	**Density ($kg\ m^{-3}$)**	**Heat capacity ($J\ kg^{-1}\ °C^{-1}$)**	**Thermal conductivity ($W\ m^{-1}\ °C^{-1}$)**
Blood	1050.00	3617.00	0.52
Saliva	994.00	4178.00	0.60
Connective tissue	1027.00	2372.00	0.39
Fat	911.00	2348.00	0.21
Muscle	1090.00	3421.00	0.49
Nerve	1075.00	3613.00	0.49
Epithelium (Skin)	1109.00	3391.00	0.37
Metal	7850.00	475.00	44.5

References

1. IT'IS Foundation. Tissue Database [Internet]. [cited 2025 Apr 30]. Available from: https://itis.swiss/virtual-population/tissue-properties/database/

Table S2. Regression coefficients for detachment force. Model 2 expands model 1 by including a squared term for metal temperature.

	Model 1			Model 2		
**Characteristic**	**Beta**	**95% CI[1]**	**p[2]**	**Beta**	**95% CI[1]**	**p[2]**
(Intercept)	6.1	-0.4, 13.6	0.067	0.2	-5.5, 5.8	0.956
Detachment method						
Gradual	—	—		—	—	
Rapid	9.3	6.2, 12.4	<0.001	9.1	6.1, 12.2	<0.001
Tongue region						
Apex	—	—		—	—	
Basis	4.3	1.5, 7.2	0.003	4.5	1.7, 7.3	0.002
Metal temperature, °C	0.45	0.26, 0.63	<0.001	45.9[3]	27.2, 64.7	<0.001
Metal temperature squared, °C	—	—	—	-29.4[3]	-47.6, -11.1	0.002
Contact time, s	1.3	0.68, 1.94	<0.001	1.3	0.67, 1.89	<0.001

[1] Wald's method. [2] Satterthwaite's method. [3] Polynomial terms are transformed by the *poly()* function in R for methodological reasons. Corresponding raw coefficients are -0.47 and -0.03 for the linear and squared term, respectively. Abbreviation: CI = Confidence Interval.

Table S3. Odds ratios for sustaining avulsion or cold injury of the tongue after contact with a cold metal lamp post.

	Avulsion injury			Cold injury		
**Characteristic**	**log(OR)**	**95% CI**	**p**	**log(OR)**	**95% CI**	**p**
(Intercept)	2.7	0.99, 4.5	0.002	-0.75	-2.6, 1.1	0.4
Metal temperature, °C	0.15	0.09, 0.21	<0.001	-0.30	-0.45, -0.14	<0.001
Contact time, s	-0.09	-0.25, 0.07	0.3	0.12	-0.14, 0.37	0.4
Detachment method						
Gradual	—	—		—	—	
Rapid	-0.12	-0.89, 0.65	0.8	—	—	
Tongue region						
Apex	—	—		—	—	
Basis	0.42	-0.30, 1.1	0.3	0.18	-1.1, 1.5	0.8

Abbreviations: CI = Confidence Interval, OR = Odds Ratio.

Table S4. Parameters used to model generalized adult and pediatric tongues.

Parameter	Adult	Child
Metal thickness, mm	4.6	4.6
Saliva thickness, mm	0.1	0.1
Epithelial thickness, mm	0.47	0.33
Connective tissue thickness, mm	1	0.7
Fat layer, mm	6.76	4.7

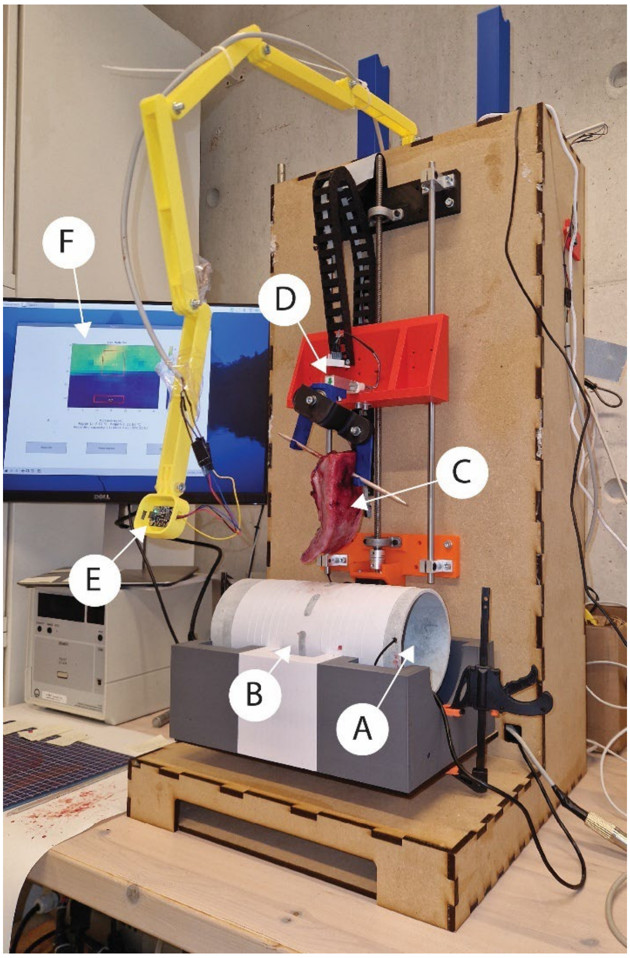

Figure S1. Overview of the experimental setup. (A) Section of a metal lamp post. (B) Template with standardized opening to ensure consistent contact area. (C) Porcine tongue. (D) Load cell mounted on a motorized platform for controlled detachment and force measurement. A second load cell was installed beneath the lamp post section. (E) Infrared (IR) camera for thermal monitoring. (F) Live thermal image from the IR camera, showing the heated tongue and the cold metal surface.

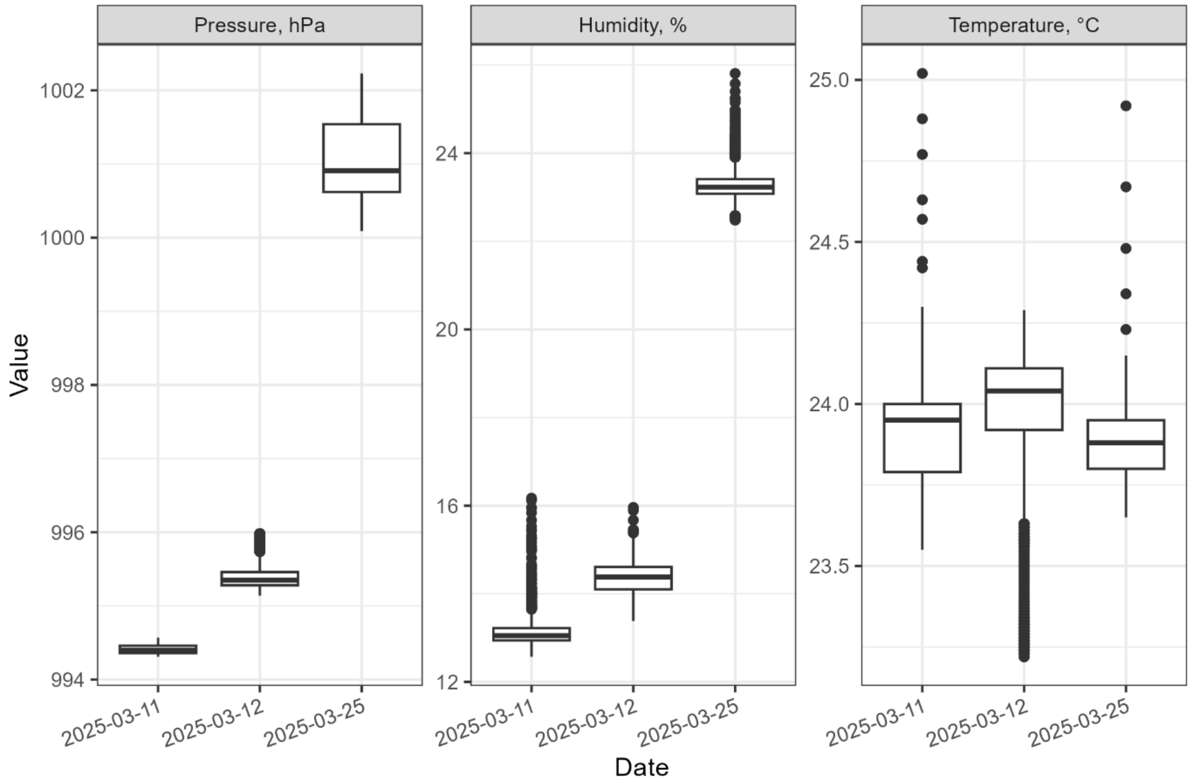

Figure S2. Distribution of ambient variables during the days of experimentation.

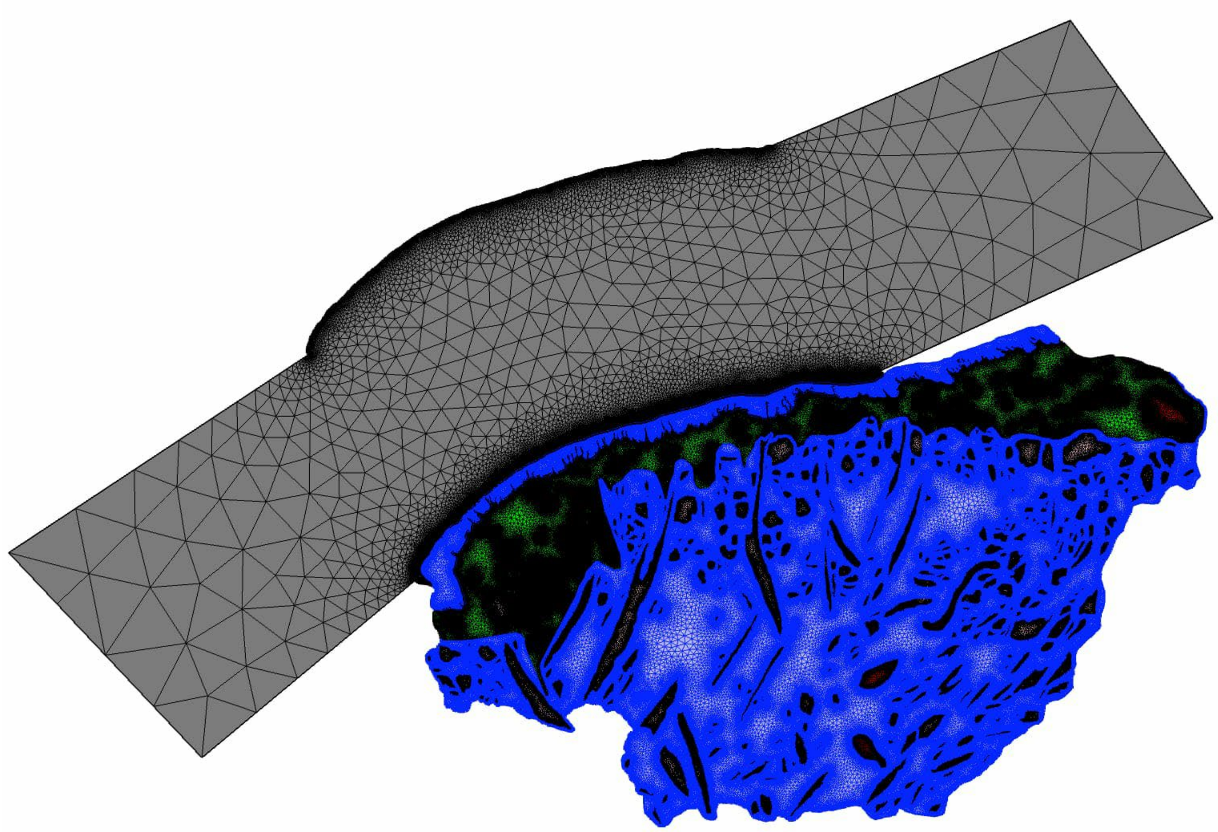

Figure S3. Mesh geometry used for the simulation model, with the metal plate shown in grey and the tongue shown in blue. Note that, because histological sections were used to construct the tongue models, the geometry implies that the metal pipe conforms to the tongue shape rather than vice-versa. This effect was minimized when a simplified tongue model was considered. The results were similar for both models, indicating that the rough metal surface has no significant impact on the results.

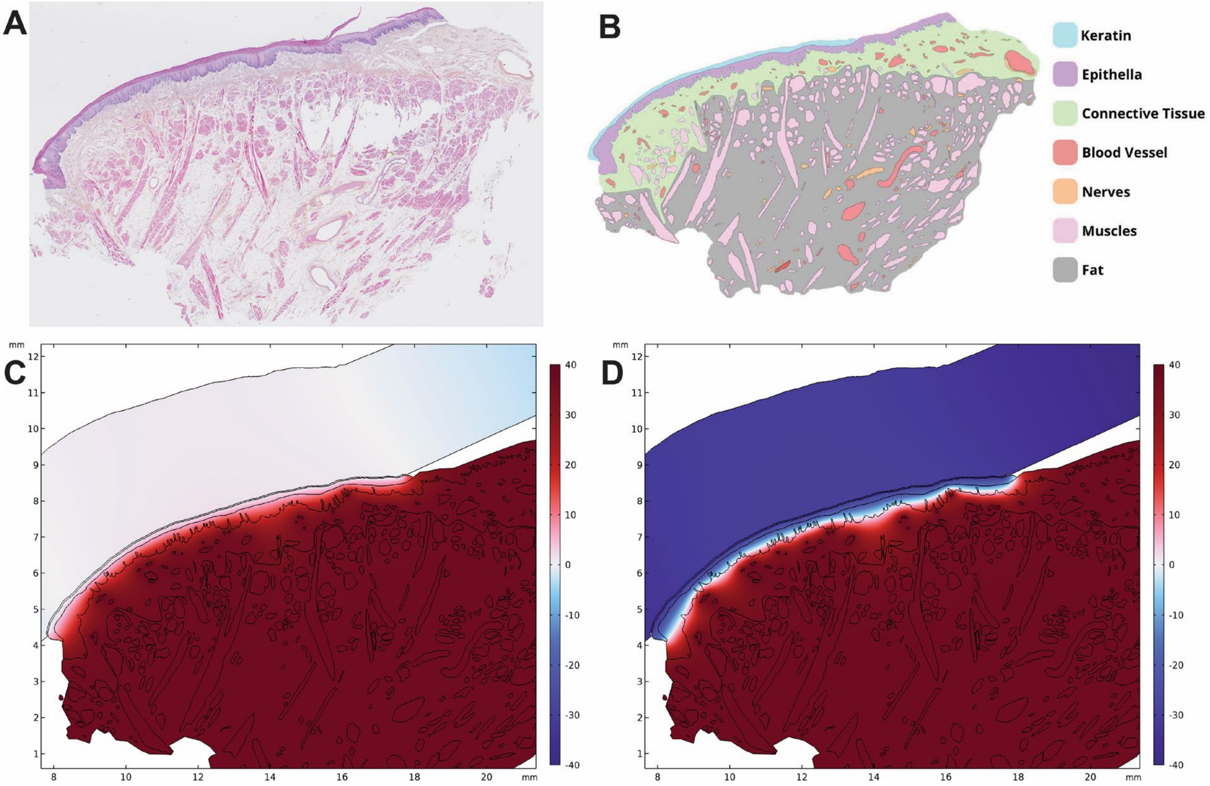

Figure S4. A histological image of an adult human tongue (A) was segmented into tissue components (B) and used to simulate tissue temperature distributions after 7 seconds of contact with metal surfaces initially at -4 °C (C) and -40 °C (D).

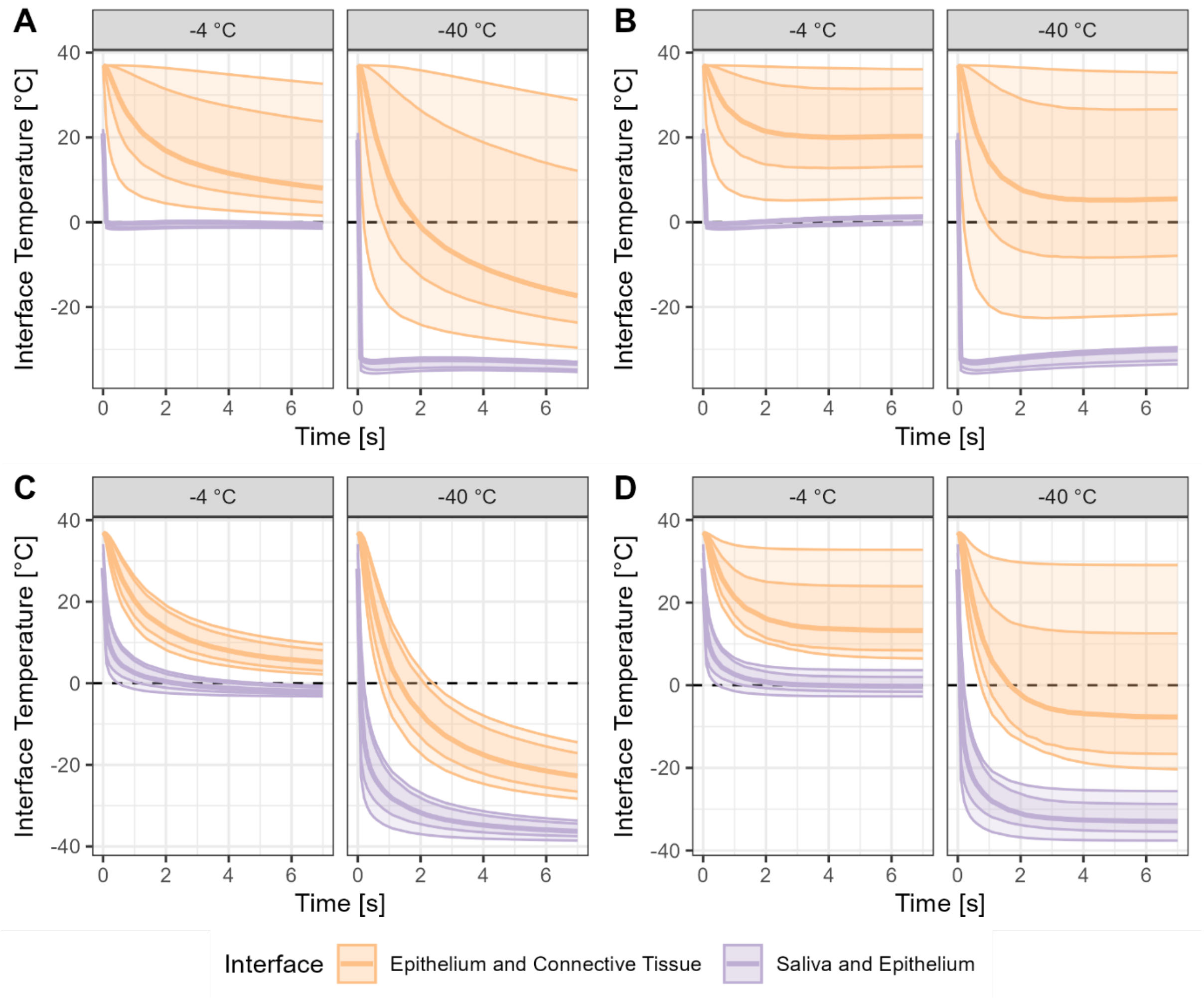

Figure S5. Temperature distribution across various tissue interfaces in (A, B) a segmented adult human tongue model and (C, D) a predictive child tongue model. Panels on the left (A, C) show simulations without blood flow, while panels on the right (B, D) include simulated blood flow. Temperatures were acquired at multiple points along the interfaces. Bold lines show the median temperatures, while shaded ribbons indicate the full range and the 10th-90th percentile intervals.

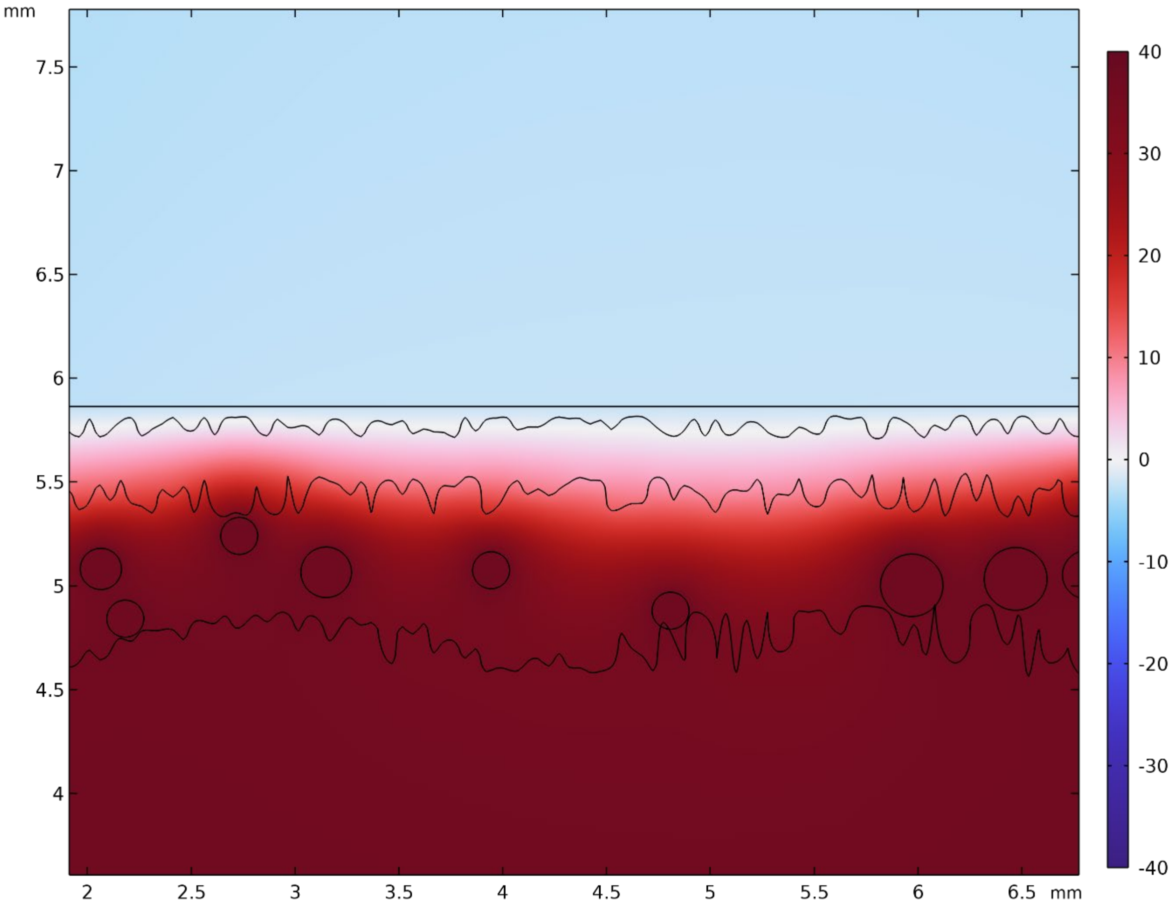

Figure S6. Simulation result of a parameterized pediatric tongue with blood flow. The simulation ran for 7 seconds, with the tongue in contact with a metal surface with an initial temperature of -4 °C. Color indicates temperature [°C].
